# Experimental Infection of North American Deer Mice with Clade I and II Monkeypox Virus Isolates

**DOI:** 10.3201/eid2904.221594

**Published:** 2023-04

**Authors:** Yvon Deschambault, Levi Klassen, Geoff Soule, Kevin Tierney, Kimberly Azaransky, Angela Sloan, David Safronetz

**Affiliations:** Public Health Agency of Canada, Winnipeg, Manitoba, Canada (Y. Deschambault, L. Klassen, G. Soule, K. Tierney, K. Azaransky, A. Sloan, D. Safronetz);; Canadian Mennonite University, Winnipeg (L. Klassen);; University of Manitoba, Winnipeg (D. Safronetz)

**Keywords:** mpox, monkeypox, MPXV, viruses, deer mouse, experimental infection, zoonoses, Canada

## Abstract

The global spread of monkeypox virus has raised concerns over the establishment of novel enzootic reservoirs in expanded geographic regions. We demonstrate that although deer mice are permissive to experimental infection with clade I and II monkeypox viruses, the infection is short-lived and has limited capability for active transmission.

Monkeypox virus (MPXV; genus *Orthopoxvirus*, *Poxviridae*), which causes mpox disease, is a zoonotic pathogen that is endemic in Central Africa (clade I) and Western Africa (clade II) (*1*). In mid-May 2022, the World Health Organization first reported an increasing number of mpox cases in nonendemic countries, most of which had no established travel links to endemic regions (*2*). By October 2022, the outbreak encompassed >100 countries with reported confirmed mpox cases (*3*). 

The global spread of MPXV outside of regions in which this virus was known to be endemic raises concerns over reverse zoonotic events resulting in the establishment of novel wildlife reservoirs. Small mammals, including rodents, have previously been implicated as enzootic reservoirs of MPXV. In North America, studies have shown that prairie dogs are susceptible to MPXV infection and may serve as a potential reservoir, but data on other wild rodents are limited (*4*). *Peromyscus* species rodents have an extensive and geographically diverse host range spanning most regions across North America and are well-established reservoirs for several zoonotic pathogens (*5*).

We evaluated the competency of deer mice (*Peromyscus maniculatus rufinus*) as a potential zoonotic reservoir for MPXV by using representative isolates from both clades. We infected groups of 12 adult (>6 weeks of age) deer mice with 1 of 3 MPXV isolates through intranasal instillation. The isolates included a clade II human isolate from the 2022 outbreak (MPXV/SP2833) (challenge dose 10^6^ PFU); a second clade II virus isolated directly from a North American prairie dog (USA-2003) (challenge dose 10^6^ PFU); and a historical clade I isolate (MPXV/V79-1-005) (challenge dose 10^4^ PFU). For each virus preparation, we administered the maximum challenge dose based on titration on Vero cells. On days 4 and 10 postinfection, we euthanized 3 male and 3 female mice and collected selected solid organs for analysis of viral titers using molecular assays targeting of envelope protein gene (B6R) (*6*) and infectious viral quantification assays. In addition, we collected oral and rectal swab specimens and tested them similarly to assess the potential for shedding.

We conducted animal studies in accordance with the Canadian Council of Animal Care guidelines and following an animal use document approved by an institutional Animal Care and Use Committee, in a Biosafety Level 4 laboratory of the Public Health Agency of Canada. We conducted fully validated molecular assays in accordance with Public Health Agency of Canada special pathogens diagnostic procedures.

Throughout the course of the study, we observed no obvious signs of disease in any of the infected deer mice. We did not record daily weights because of the requirement for anesthetizing animals before any hands-on manipulation. Analysis of tissue samples from mice infected with the 2022 Canada isolate (MPXV/SP2833) revealed limited and sporadic spread of MPXV beyond the sites of inoculation (nasal turbinates and lungs) (Table). By comparison, USA-2003 appeared to disseminate beyond the respiratory tract, resulting in uniform detection of MPXV DNA in liver and spleen specimens collected at 4 days postinfection (dpi). The clade I virus (MPXV/V79-1-005) yielded results more similar to those for USA-2003; nasal turbinate, lung, liver and spleen samples were positive at 4 dpi. By day 10 dpi, organ specimens from most mice across the 3 infection groups were trending toward clearance (Table). Infectious titers conducted on lung and nasal turbinate specimens collected at both timepoints from the 3 challenge groups corroborated these findings and demonstrated decreasing viral titers between the 2 timepoints (Figure). 

**Table:** Summary of PCR results of selected organs collected from deer mice experimentally infected with 3 different MPXV isolates*

Tissue	Sex	MPXV/SP2833			USA-2003			MPXV/V79-1-005	
		4 dpi	10 dpi		4 dpi	10 dpi		4 dpi	10 dpi
Nasal turbinate	M	3/3 (100)	1/3 (33)		3/3 (100)	3/3 (100)		2/3 (67)	2/3 (67)
	F	3/3 (100)	3/3 (100)		3/3 (100)	2/3 (67)		3/3 (100)	2/3 (67)
	Total	6/6 (100)	4/6 (67)		6/6 (100)	5/6 (83)		5/6 (83)	4/6 (67)
Lung	M	3/3 (100)	0/3 (0)		3/3 (100)	3/3 (100)		3/3 (100)	2/3 (67)
	F	3/3 (100)	1/3 (33)		3/3 (100)	1/3 (33)		3/3 (100)	2/3 (67)
	Total	6/6 (100)	1/6 (17)		6/6 (100)	4/6 (67)		6/6 (100)	4/6 (67)
Heart	M	1/3 (33)	0/3 (0)		2/3 (67)	0/3 (0)		1/3 (33)	0/3 (0)
	F	0/3 (0)	0/3 (0)		0/3 (0)	0/3 (0)		0/3 (0)	0/3 (0)
	Total	1/6 (17)	0/6 (0)		2/6 (33)	0/6 (0)		1/6 (17)	0/6 (0)
Liver	M	0/3 (0)	0/3 (0)		3/3 (100)	0/3 (0)		2/3 (67)	0/3 (0)
	F	0/3 (0)	0/3 (0)		3/3 (100)	2/3 (67)		3/3 (100)	0/3 (0)
	Total	0/6 (0)	0/6 (0)		6/6 (100)	2/6 (33)		5/6 (83)	0/6 (0)
Spleen	M	1/3 (33)	0/3 (0)		3/3 (100)	1/3 (33)		2/3 (67)	0/3 (0)
	F	0/3 (0)	0/3 (0)		3/3 (100)	1/3 (33)		3/3 (100)	0/3 (0)
	Total	1/6 (17)	0/6 (0)		6/6 (100)	2/6 (33)		5/6 (83)	0/6 (0)
Small intestine	M	1/3 (33)	0/3 (0)		1/3 (33)	0/3 (0)		0/3 (0)	0/3 (0)
	F	0/3 (0)	0/3 (0)		2/3 (67)	0/3 (0)		0/3 (0)	0/3 (0)
	Total	1/6 (17)	0/6 (0)		3/6 (50)	0/6 (0)		0/6 (0)	0/6 (0)
Oral swab	M	3/3 (100)	1/3 (33)		3/3 (100)	1/3 (33)		1/3 (33)	0/3 (0)
	F	2/3 (67)	1/3 (33)		3/3 (100)	2/3 (67)		1/3 (33)	0/3 (0)
	Total	5/6 (83)	2/6 (33)		6/6 (100)	3/6 (50)		2/6 (33)	0/6 (0)
Rectal swab	M	3/3 (100)	1/3 (33)		3/3 (100)	2/3 (67)		0/3 (0)	1/3 (33)
	F	2/3 (67)	1/3 (33)		1/3 (33)	2/3 (67)		0/3 (0)	0/3 (0)
	Total	5/6 (83)	2/6 (33)		4/6 (67)	4/6 (67)		0/6 (0)	1/6 (17)

*Values are no. (%). dpi, days postinfection; MPXV, monkeypox virus.

**Figure:**
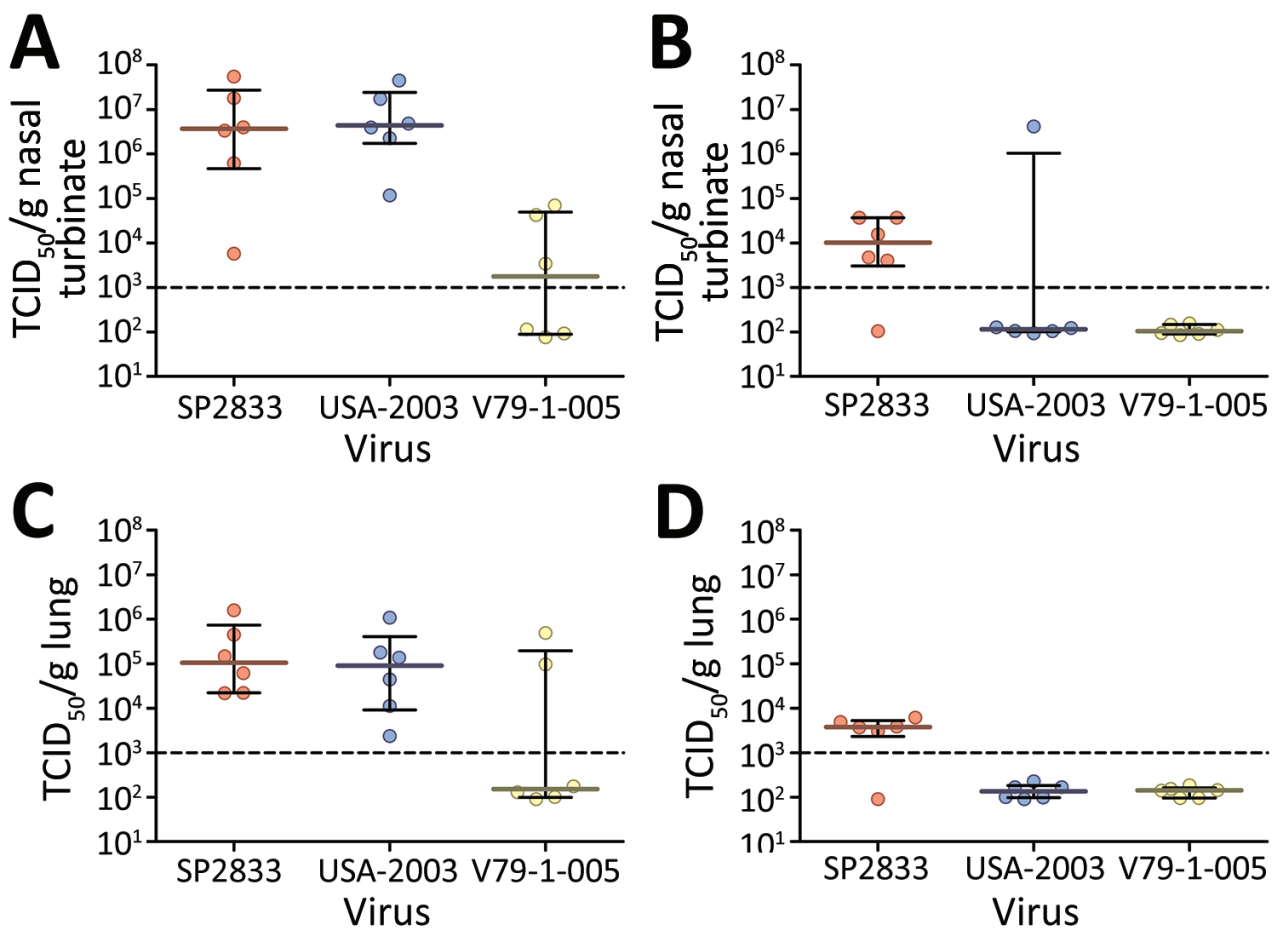
Monkeypox virus infectious titers from lung and nasal turbinate samples from experimentally infected deer mice. Groups of 12 deer mice (6 male, 6 female) were experimentally infected with monkeypox virus isolates SP2833, USA-2003 (both clade II), or V79-1-005 (clade II) through the intranasal route. Lung and nasal turbinates were collected from 3 male and 3 female rodents at days 4 and 10 postinfection and infectious titers assessed using standard tissue culture methods. Shown are the infectious titers for individual specimens (dots) or median values (solid lines) and interquartile ranges (error bars) for nasal turbinate specimens collected at day 4 (A) and day 10 (B) postinfection and lung specimens collected at day 4 (C) and day 10 (D) postinfection. Dotted line represents the lower limit of detection of the assay. TCID_50_, median tissue culture infectious dose.

Of note, the clade I virus did not achieve high titers in either organ, even when analyzed at 4 dpi. Although this finding may suggest the MPXV/V79-1-005 isolate does not replicate as efficiently in deer mice, the apparent low viral titers observed may be attributable to the lower inoculum dose. A similar challenge dose of this strain resulted in lethal infection in CAST/EiJ mice (*7*). Further, subsequent cell culture propagations of MPXV/V79-1-005 resulted in similar titers as the clade II isolates used previously, suggesting that all 3 replicate to a similar extent on Vero cells. Nevertheless, follow-up studies with other clade I viruses are warranted.

We collected oral and rectal swab specimens to assess shedding and the potential for transmission of MPXV from infected deer mice. Overall, shedding, as suggested by the presence of MPXV DNA in swab extracts, was readily detectable in deer mice inoculated with either clade II virus at day 4, but we noted decreasing levels of positivity by day 10. Shedding of MPXV/V79-1-005 (clade 1) was far less than that of either of the clade II viruses we evaluated (Table).

Our study suggests that these rodents may support a short-term but abortive infection with at least clade II MPXV isolates, although with limited capacity to spread. Given the short duration of infection, these animals probably do not represent a viable enzootic reservoir for MPXV. Further studies should be conducted on other rodents in North America and Europe to assess their competency as vectors or reservoirs of MPXV. Particular interest should be given to *Rattus* species rodents that may frequently come into contact with medical waste containing viable MPXV.
